# Development of a Highly Sensitive Humidity Sensor Based on a Piezoelectric Micromachined Ultrasonic Transducer Array Functionalized with Graphene Oxide Thin Film

**DOI:** 10.3390/s18124352

**Published:** 2018-12-10

**Authors:** Changhe Sun, Qiongfeng Shi, Mahmut Sami Yazici, Chengkuo Lee, Yufei Liu

**Affiliations:** 1 Centre for Intelligent Sensing Technology, College of Optoelectronic Engineering, Chongqing University, Chongqing 400044, China; chsun@cqu.edu.cn; 2 Department of Electrical and Computer Engineering, National University of Singapore, 4 Engineering Drive 3, Singapore 117583, Singapore; eleshiq@nus.edu.sg (Q.S.); sami.yazici@u.nus.edu (M.S.Y.); 3 Center for Intelligent Sensors and MEMS, National University of Singapore, E6 #05-11F, 5 Engineering Drive 1, Singapore 117608, Singapore; 4 Key Laboratory of Optoelectronic Technology & Systems (Chongqing University), Ministry of Education, Chongqing 400044, China; 5 Collaborative Innovation Center for Brain Science, Chongqing University, Chongqing 400044, China

**Keywords:** piezoelectric micromachined ultrasonic transducer, humidity sensor, graphene oxide, array structure, high sensitivity

## Abstract

A novel relative humidity sensor that is based on a linear piezoelectric micromachined ultrasonic transducer (pMUT) array was proposed and microfabricated for high sensitivity, fast response, and good stability. The humidity-sensitive graphene oxide (GO) film was deposited on the pMUT array with a facile drop-casting method and characterized by scanning electron microscope (SEM), atomic force microscope (AFM), and Fourier transform infrared spectrum (FTIR). With the humidity level ranging from 10% to 90% RH, the sensor exhibited a high sensitivity of 719 Hz/% RH and an extremely high relative sensitivity of 271.1 ppm/% RH. The humidity-sensing results also showed good short-term repeatability and long-term stability, fast response and recovery, and low hysteresis. Moreover, the temperature coefficient of frequency (TCF) of the present humidity sensor was investigated and it could be easily compensated owing to the pMUT array structure design. This work confirmed that the GO functionalized pMUT is an excellent candidate in humidity detection and it may enable many potential applications, such as ultrasensitive mass detection and simultaneous detection of multiple parameters.

## 1. Introduction

Miniaturized humidity sensors enabled through micro-/nano-fabrication technologies have gained extensive attention due to distinctive advantages of small size, low cost, fast response, high sensitivity, and high stability. These micromachined humidity sensors have great potential for portable electronic systems and they play crucial roles in a wide range of applications, such as environmental monitoring, industrial process control, agricultural production, and medical treatments [1,2]. In general, there are a variety of humidity sensing principles, mainly including optical methods [3,4], electrical methods (resistance [5], capacitance [6], and impedance [7]), and resonant mechanical methods (surface acoustic wave (SAW) resonator [8], film bulk acoustic resonator (FBAR) [9], quartz crystal microbalance (QCM) [10], micro-/nano-cantilever [11], and capacitive micromachined ultrasonic transducer (cMUT) [12]). When compared with other humidity sensing technologies, cMUT as an emerging ultrasound generator and detector proposed by Kuri-Yakub group has been proven to be an ultrasensitive and powerful approach for mass load detection, combining low effective mass and quite large active surface [13]. Benefitting from the extremely high mass sensitivity at the zeptogram (zg) scale, the cMUT-based chemical sensor with a selectively sensitive layer exbibits an excellent sensing performance towards water vapor [12]. However, such good performance relies heavily on very narrow cavity (~40 nm) and high bias voltage (~50 V), which largely increases the process difficulty and fabrication cost and further limits its application fields. Similar to the array structure and resonant operation mode of cMUT, piezoelectric micromachined ultrasonic transducer (pMUT) operating at only several volts without a specially designed cavity can also be easily functionalized for chemical and physical sensing by coating the selective sensing material on the device surface. Due to its inherent mass-sensing property, multi-sensor array configuration, and manufacturing advantages when compared with the existing QCM, SAW, and FBAR piezoelectric sensors, the pMUT array is highly promising and much advantageous to provide high humidity sensitivity. However, the humidity sensor that is based on the pMUT has not been investigated yet to our best knowledge.

It is well known that humidity-sensing properties, such as sensitivity, response, and stability are largely affected by the selected sensitive thin film. Until now, various kinds of humidity-sensing materials have been employed, including metal oxides [14,15], ceramics [16,17], carbonic materials [18,19], polymers [20,21], and their composites [22,23,24], etc. Among these materials, graphene oxide (GO), as a typical chemical derivative of graphene, has attracted great interest due to its layered honeycomb structure, containing abundant reactive oxygen functional groups (carboxyl, hydroxyl, and epoxy groups) in each layer [25]. The existence of these hydrophilic oxygen groups makes GO an excellent candidate for humidity detection. Previous studies have demonstrated high sensitivity, fast response and little hysteresis of GO-based resonators [19,26]. Besides, owing to its electrical insulation characteristic, GO can be directly deposited on the electrodes.

In this work, a linear pMUT array based humidity sensor functionalized with GO sheets were proposed and fabricated with the microelectromechanical system (MEMS) technology. The GO thin film was directly deposited on the microfabricated pMUT array with a simple and facile drop-casting method and was characterized by scanning electron microscope (SEM), atomic force microscope (AFM), and Fourier transform infrared spectrum (FTIR). The humidity sensing characteristics of developed sensors, such as response/recovery, hysteresis, stability, and temperature effect, were investigated and discussed. Impedance and phase curves at different RH levels were also used to characterize the GO coated pMUT humidity sensor. Last, the sensing mechanism of the present sensor was analysed. This research demonstrates the potential of the pMUT based humidity sensor for highly sensitive mass detection in a wide range of applications.

## 2. Experimental

### 2.1. Design and Fabrication of pMUT Humidity Sensor

**Figure 1:**
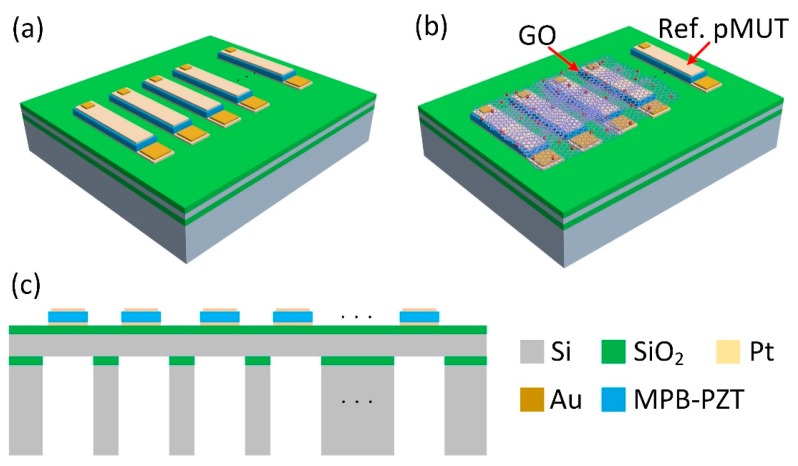
Schematic structure of (**a**) the piezoelectric micromachined ultrasonic transducer (pMUT) array and (**b**) pMUT-based humidity sensor, (**c**) cross-sectional view of the pMUT array.

The pMUT based humidity sensor contains a pMUT linear array that was fabricated on a released silicon-on-insulator (SOI) wafer and a GO thin film deposited on the pMUT array. The schematic structures of the pMUT array and pMUT based humidity sensor are illustrated in Figure 1a,b. The pMUT linear array consists of 15 rectangular pMUT elements with a good frequency consistency, some of which are coated with GO film for humidity sensing, while others are uncoated for reference. The pMUT is designed with the morphotropic phase boundary composite lead zirconate titanate (Zr/Ti = 52/48, MPB-PZT) piezoelectric material. The dimensions of piezoelectric membrane and the underlying cavity are 120 μm (Width) × 500 μm (Length) × 1.9 μm (Thickness) and 160 μm (Width) × 550 μm (Length) × 400 μm (Height), respectively. The cross-sectional view of the pMUT array is shown in Figure 1c, which contains 10 μm Si/1 μm SiO_2_/200 nm Pt/1.9 μm MPB-PZT/200 nm Pt.

The fabrication process started with an n-type SOI wafer with 10 μm thick device layer. A 1 μm SiO_2_ layer was deposited on the SOI wafer for electrical insulation, and then 200 nm Pt/10 nm Ti thin films were deposited by direct current (DC) magnetron sputtering and patterned as the bottom electrodes by Ar ions. After that, a layer of 1.9 μm MPB-PZT was formed using the sol-gel process and patterned through wet-etching. Next, another 200 nm Pt/10 nm Ti thin films were deposited and patterned as top electrodes. Last, the Si substrate was etched by deep reaction-ion etching (DRIE) to release the membrane. The 100 nm Au was formed as wire bonding pads. The as-fabricated pMUT array was then functionalized by drop-casting of diluted GO dispersions with concentration of 1 mg/mL. The original GO dispersions was prepared by the modified Hummers’ method and supplied from Suzhou Tanfeng Graphene Technology Co., Ltd. (Suzhou, China). After deposition, the prepared pMUT humidity sensors were placed into the oven and heated at 60 °C for 48 h. Before the experiments, all pMUT humidity sensors under the test were packaged and wire-bonded in the double in-line package (DIP) holders. The polydimethylsiloxane (PDMS) was applied to protect the bonding wires from damage during chip picking up and seal the backside released cavity for eliminating the interferences of moistures and gases that are trapped in the cavity when exposed to humidity conditions.

### 2.2. Working Principle

The basic resonant structure of one rectangular pMUT element is a PZT/Si layered rectangular membrane with fully clamped boundaries. The resonant characteristics are mainly determined by the material properties and the dimensions of the vibrating membranes. The fundamental flexural-mode operating frequency for a rectangular membrane is derived by Ref. [27]

(1) $$f_{0}=\frac{0.494t}{W^{2}}\sqrt{\frac{E}{\rho \left(1-\nu^{2}\right)}\left[1+\frac{2}{3}{\left(\frac{W}{L}\right)}^{2}+{\left(\frac{W}{L}\right)}^{4}\right]}$$

where *t*, *L*, *W*, *E*, *ρ*, and *ν* are the thickness, length, width, Young’s modulus, Poisson’s ratio, and density of the rectangular membrane. It is shown that the operating frequency of the pMUT is proportional to the Young’s modulus and inversely proportional to the density. Therefore, coating a selectively sensitive thin film on the pMUT surface could shift the operating frequency upward or downward, which is mainly dependent on the material properties and thickness of the deposited layer. However, the physisorption or chemisorption of the analyte molecules on the sensitive thin film would result in a frequency decrease due to the mass or density change, well-known as the mass-loading effect. The frequency shift Δ*f* of the developed pMUT humidity sensor can be estimated by the following equation [12]:

(2) $$\Delta f=-\frac{1}{2}f_{0}\times \frac{\Delta m}{m}$$

where *m* and Δ*m* are the mass of the effective vibration membrane and mass change after absorption of water molecules, respectively. According to the mechanical resonant frequency Expression (1) and the dimensions, the mass sensitivity per unit area of the designed pMUT is estimated at 16 ag/Hz/μm^2^.

### 2.3. Characterization and Measurement

**Figure 2:**
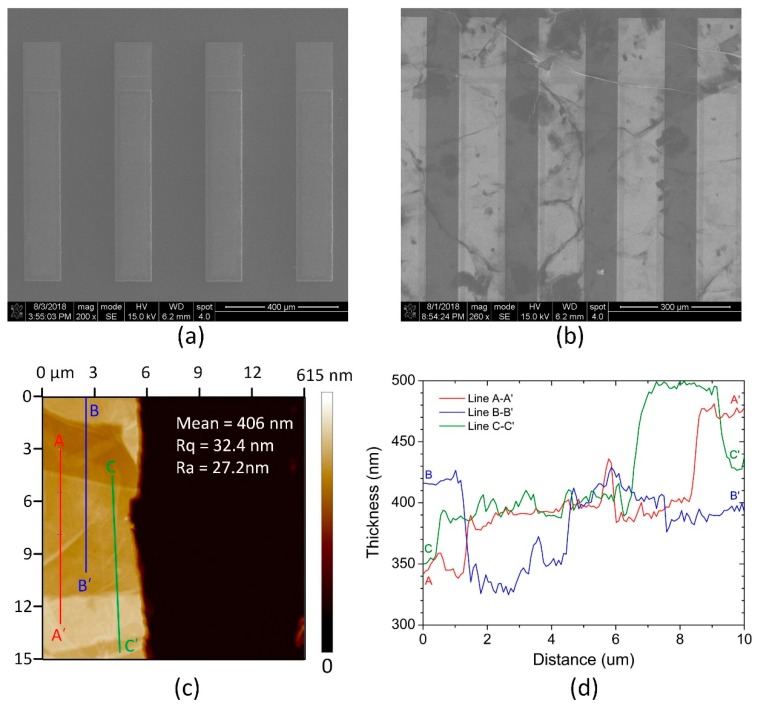
Scanning electron microscope (SEM) images of the pMUTs (**a**) before coating the graphene oxide (GO) film and (**b**) after coating the GO film, (**c**) atomic force microscope (AFM) image, and (**d**) height profile analysis of the deposited GO film.

The surface morphologies of the pMUT humidity sensor before and after coating GO film were characterized by SEM operated at 15 kV, as shown in Figure 2a,b. To investigate the thickness and uniformity of the GO film, the same volume and concentration of GO dispersions was dropped on the surface of a flat Si wafer and heated at 60 °C for 24 h. The AFM analysis of the deposited GO film is shown in Figure 2c,d. It is obviously seen that the resultant GO thin film has a layered and wrinkled structure, which is associated with the exfoliation process during the GO film preparation. The average thickness of the GO film is about 406 nm, with the surface roughness *Rq* and *Ra* of 32.4 nm and 27.2 nm, respectively.

**Figure 3:**
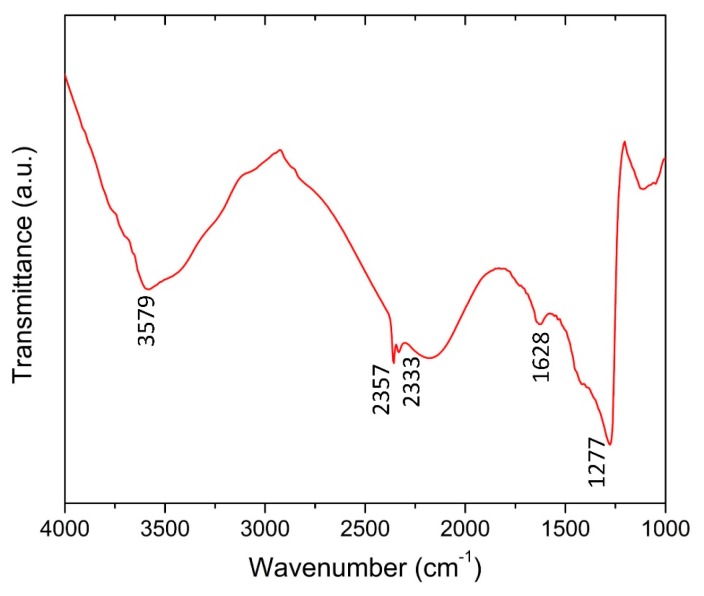
Fourier Transformed Infrared (FTIR) spectrum of the GO thin film.

Figure 3 shows the Fourier Transformed Infrared (FTIR) spectrum of the GO film in the range 4000–1000 cm^−1^ obtained by a high-resolution FTIR microscope at the transmission mode. Two peaks appeared at 2357 cm^−1^ and 2333 cm^−1^ due to the formation of unique chemical species in the presence of CO_2_ [28]. A characteristic absorption peak at 1628 cm^−1^ suggests the presence of carboxyl groups (C=O stretching vibrations). The strong and broad valley at 3579 cm^−1^ is correlated to the –OH stretching vibrations.

**Figure 4:**
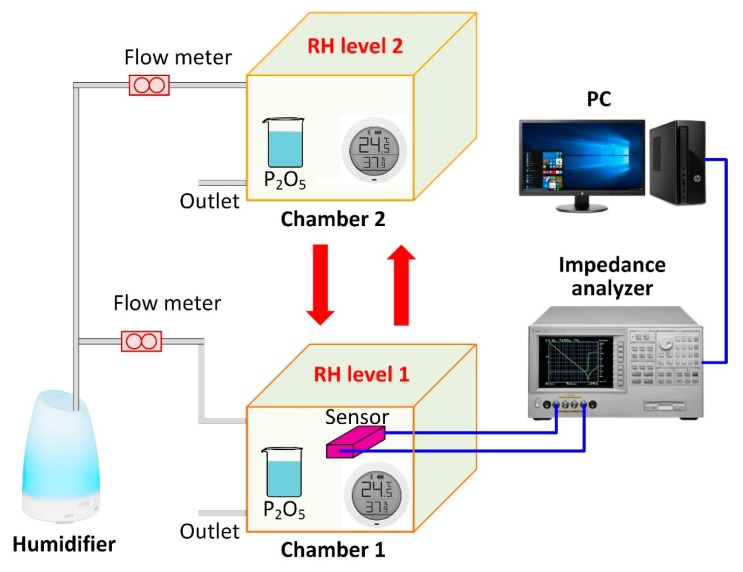
Experimental setup for humidity sensing property measurement.

The experimental setup for humidity-sensing property measurement is illustrated in Figure 4. The pMUT humidity sensors were placed in one of two sealed plastic chambers. The P_2_O_5_ desiccant and humidifier filled with deionized (DI) water were used together to achieve humidity levels from 10% RH to 90% RH with a 10% RH interval. The relative humidity in the test chambers was manually changed by adjusting the flow rate of the evaporative moistures and it was recorded in real time by a precise relative humidity meter. A precision impedance analyser (Agilent 4294A, Agilent Technologies Inc., Singapore) was used to measure the resonant frequency of the pMUT humidity sensors and the data were transmitted to a personal computer (PC) for saving and off-line processing. The temperature in the chamber was kept constant at 24 ± 0.5 °C to eliminate the interference from temperature changes. A Xiaomi Mijia bluetooth humidity temperature sensor (Xiaomi Inc., Beijing, China) was also applied to track both the humidity and temperature values per second. During the experiments, the sensors were placed in one chamber first to obtain the response at one humidity level, and then were rapidly transferred into the other chamber to obtain the response at another humidity level. In this way, the humidity-sensing response and hysteresis characteristics could be explored by changing the relative humidity in two chambers from 10% RH to 90% RH and then back to 10% RH.

## 3. Results and Discussion

### 3.1. Performance of pMUT without GO Film

**Figure 5:**
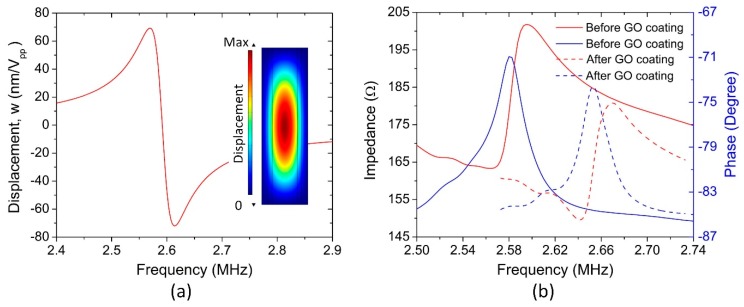
(**a**) Simulated displacement response of the pMUT and the associated mode shape (**b**) measured impedance and phase curves of the pMUT before and after GO coating.

The finite element method (FEM) simulation that was based on COMSOL Multiphysics v5.2a was first employed to study the resonant characteristics of the pMUT. Figure 5a shows the predicted response spectrum with a fundamental frequency of 2.58 MHz and a displacement sensitivity of 80 nm/V_pp_. Subsequently, both the impedance and phase curves of the pMUT humidity sensors were measured before and after coating the GO thin film, as plotted in Figure 5b. By comparison with the uncoated case, it is found that there is a slight increase in the resonant frequency, which might be caused by an increase in the mechanical stiffness of the pMUT after depositing GO film.

### 3.2. Humidity-Sensing Properties

**Figure 6:**
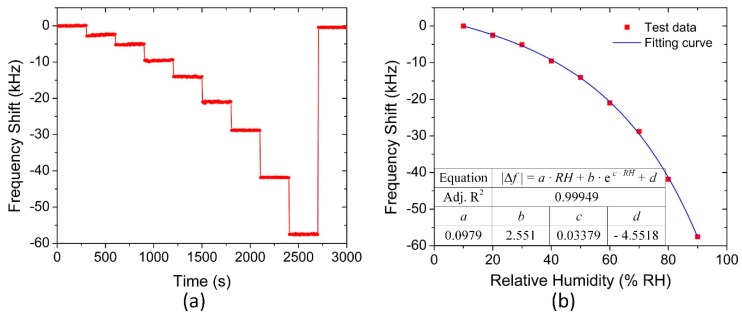
(**a**) Steady frequency response of the pMUT humidity sensor and (**b**) the relationship between the frequency shift and relative humidity levels.

The pMUT humidity sensors were tested in the well-sealed plastic chamber with the relative humidity increasing from 10% to 90% RH. The frequency shift to different humidity levels is shown in Figure 6a,b. When the pMUT sensors were exposed to the low humidity condition, there is an approximately linear relationship between the frequency shift and the humidity levels. However, when the humidity is continuously increased to a high RH level, a nonlinear decrease in the resonant frequency can be observed, which is in good accordance with the previous reports [19,26]. The whole absorption process of water molecules in the GO film can be divided into two steps: superficial adsorption at the low humidity level and volumetric adsorption at the high humidity level. Thus, the frequency shift Δ*f* with respect to the relative humidity levels can be fitted and described by the following expression:

(3) $$\left|\Delta f\right|=a\cdot RH+b\cdot e^{c\cdot RH}+d$$

where *a*, *b*, *c*, and *d* are the fitting coefficients and they are estimated correspondingly as 0.0979, 2.551, 0.03379, and −4.5518.

The humidity sensitivity *S* and relative humidity sensitivity *S_R_* are defined as

(4) $$S=\frac{\left|\Delta f\right|}{\Delta RH}$$

(5) $$S_{R}=\frac{S}{f_{0}}=\frac{\left|\Delta f\right|}{f_{0}\cdot \Delta RH}$$

where *f*_0_ is the fundamental resonant frequency of the pMUT humidity sensor with the GO thin film at 10% RH. Therefore, the sensitivity and relative sensitivity of the pMUT humidity sensor in the RH range of 10% to 90% RH are calculated as 719 Hz/% RH and 271.17 ppm/% RH, respectively. When compared with other resonant humidity sensors, our present pMUT sensor tends to exhibit an extremely high relative sensitivity, as summarized in Table 1.

**Figure 7:**
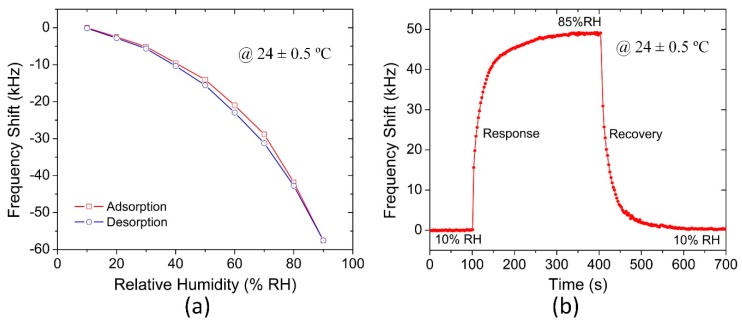
(**a**) Hysteresis curve and (**b**) dynamic response and recovery curves of the pMUT humidity sensor.

The hysteresis loops versus RH levels of the pMUT humidity sensor is shown in Figure 7a. The humidity response hysteresis is defined as the ratio of the maximum lagged frequency variation and the maximum frequency shift in the sensing range, thus the hysteresis of the sensor is calculated as 3.95% RH. The hysteretic desorption mainly resulted from the residual water molecules in the deep film. When the humidity level in the chamber returns to 10% RH, the hysteresis is nearly disappeared.

To study the dynamic response and recovery behaviours of the pMUT humidity sensor, two test chambers were kept at 10% RH and 85% RH, respectively. The sensor was manually transferred from one to the other chamber and was then transferred back. It took about 2 s to transfer the pMUT sensor. The response and recovery curves are shown in Figure 7b. The response time and the recovery time (reaching 90% of the final value) of the pMUT humidity sensor are about 78 s and 54 s, respectively. It is worth mentioning that there is an approximate linear adsorption when the frequency shift is larger than 43 kHz, which might be attributed to the unavoidable humidity fluctuation after opening and closing the chamber. Generally, the humidity would decrease by about 2% RH after quickly opening the chamber and then gradually rising by 1% RH to reach a new steady state when the chamber is closed. Therefore, the pMUT humidity sensor should be able to exhibit a shorter response time than the tested result.

**Figure 8:**
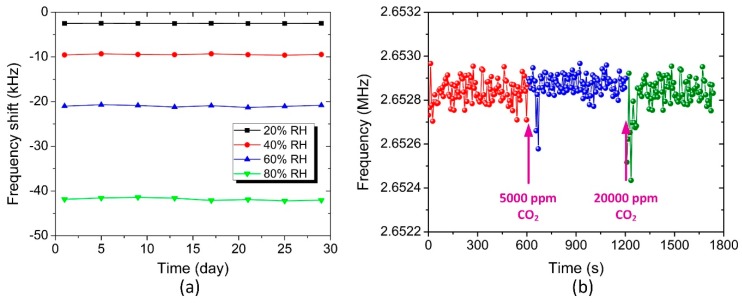
(**a**) Long-term stability of the pMUT humidity sensor at different RH levels for one month and (**b**) real time frequency response of the sensor to CO_2_ gas.

The frequency stability including short-term stability and long-term stability is an important parameter during the measurements. The short-term stability can be expressed as an Allan deviation (i.e., sigma-tau σ(τ)) to estimate the noise Δ*f* and the limit of detection (LOD) of the humidity sensor [12]. The average overlapped Allan deviation, as measured under various RH levels, is 95 Hz (1σ) for the developed pMUT humidity sensor. Thus, the humidity detection resolution of this sensor is 0.40% RH (3σ) and it is very promisingly improved by employing a higher Q-factor pMUT. The long-term stability of the pMUT humidity sensor was verified by monitoring its resonant frequency at the fixed humidity levels of 20% RH, 40% RH, 60% RH, and 80% RH per four days for one month. As illustrated in Figure 8a, the sensor has a good stability performance, with only a small variation of less than 3.2%. The fluctuations at each humidity level might be caused by the test system noise, temperature change, and uneven water molecule adsorption at the surface of the GO film. Figure 8b shows the frequency response of the pMUT humidity sensor when exposed to CO_2_ gas. It should be mentioned that during the CO_2_ sensing experiment, the relative humidity and temperature in the chamber were kept at about 10% RH and 24 ± 1 °C, respectively, to eliminate the interference from humidity and temperature changes. The CO_2_ concentration was initially kept at 0 ppm by introducing dry N_2_ gas for 10 min. The measurement results demonstrate that this sensor has no obvious response, even when exposed to CO_2_ gas with the concentration up to 20,000 ppm.

**Figure 9:**
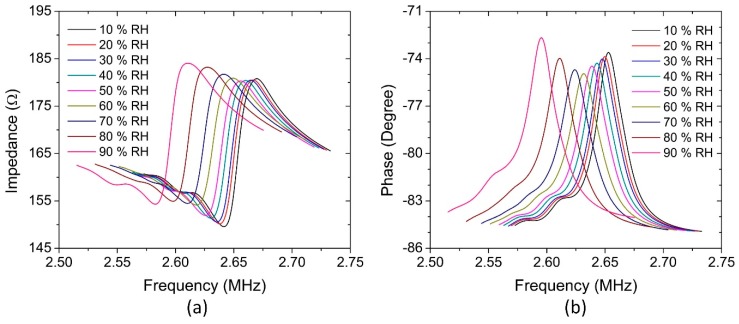
(**a**) Impedance response spectra and (**b**) phase response spectra of the pMUT humidity sensor at various RH levels.

The impedance and phase response spectra were also recorded at various RH levels, as shown in Figure 9. It can be observed that all resonant frequencies of the impedance and phase spectra are shifted downward as the RH level increases. It is also seen that the phase resonance peak has a small degradation when the relative humidity increases from 10% to 60% RH and a remarkable improvement when the humidity continuously increases to 90% RH. The change in the first stage could be caused by water molecules mass load and the viscosity increase in the sensing film, while the change in the next stage could be interpreted as the large interlayer expansion of the sensing film after the absorption of abundant moistures.

**Figure 10:**
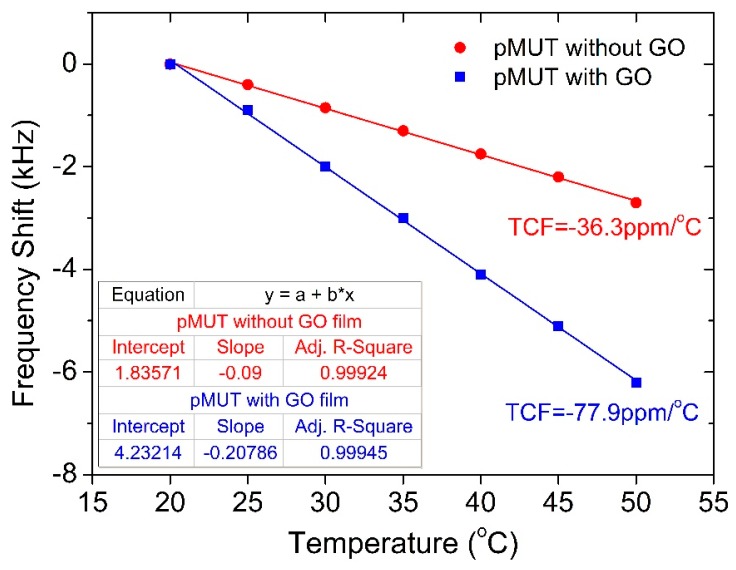
Temperature coefficients of frequency of the pMUTs with and without the GO film.

In practical applications, the environment temperature change would inevitably affect the frequency shift of the sensor and lead to an inaccurate testing result, namely the temperature effect. In this work, the temperature effect of the pMUT before and after coating the GO thin film was measured in the temperature range of 20 °C to 50 °C, as shown in Figure 10, where the humidity was kept constant at 10% RH to eliminate the interference from humidity. It is found that the resonant frequency decreases linearly as the temperature increases. The frequency dependence on temperature is characterized using the temperature coefficient of frequency (*TCF*), defined by:

(6) $$\mathrm{TCF}=\frac{1}{f_{0}}\frac{\Delta f}{\Delta T}$$

where Δ*T* is the change in temperature. The *TCF*s of the pMUTs with and without GO thin film are −36.3 ppm/°C and −77.9 ppm/°C, respectively. It is noted that such temperature impact on the resonant frequency can be easily compensated due to the perfect linear frequency-temperature relationship and the multi-pMUT array structure, which is much more advantageous than other humidity sensors. Benefiting from the linear pMUT array design, we can use some untreated pMUT elements as the reference sensor to detect the temperature change and then compensate the temperature effect of the functionalized pMUT sensors during the humidity detection. Moreover, the pMUT array also confers a potential advantage in the simultaneous detection of humidity and temperature by adopting two pMUT elements to sense them independently.

### 3.3. Sensing Mechanisim

**Figure 11:**
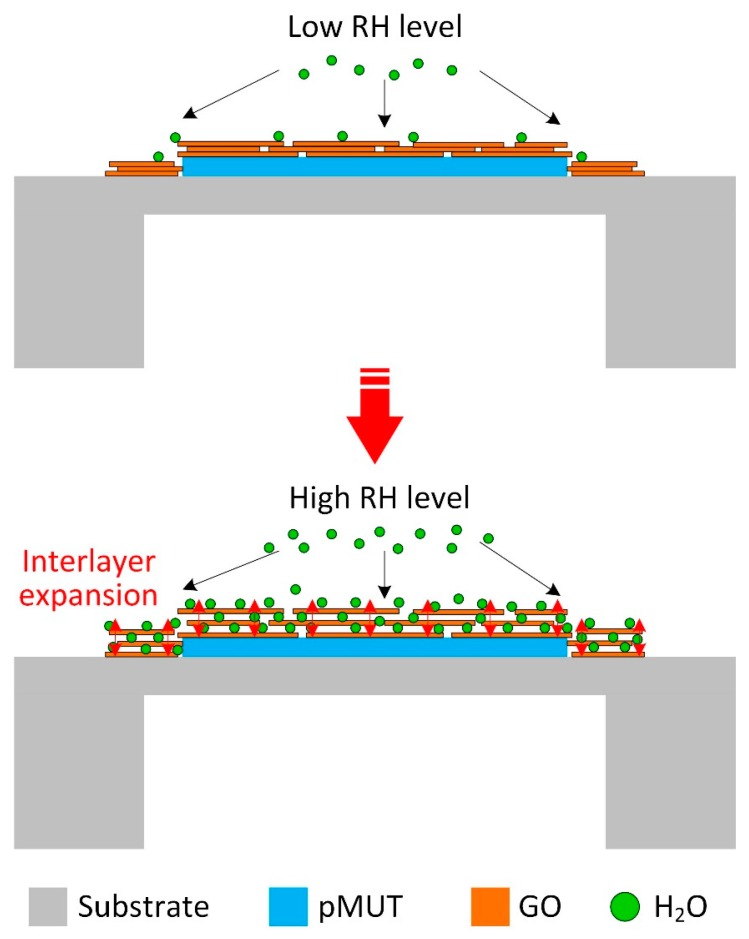
Schematic illustration of humidity-sensing mechanism of GO thin film coated pMUT.

The present humidity-sensing results indicate that the GO film functionalized pMUT is highly sensitive to water molecule adsorption, exhibiting a nonlinear frequency response and a high sensitivity of 719 Hz/% RH, as well as a high relative sensitivity of 217 ppm/% RH. A speculative mechanism explanation for these behaviours is illustrated in Figure 11. The adsorption of water molecules only occurs at the surface and edges of GO sheets, while the permeation through the GO sheets is largely restricted due to the two-dimensional (2D) honeycomb structure. For the stacked GO layers, water molecules are primarily adsorbed on the external surface though the carboxyl, hydroxyl, and epoxy groups attached at the edges of GO. With the concentration of the water molecules increasing, the water molecules migrate into the interlayer space and wander around, forming clusters bound via hydrogen bonds to oxygen-containing groups from GO and producing a slight interlayer expansion [26]. When exposed at a high RH level, a significant accumulated interlayer expansion would be produced after trapping a large amount of water molecules between the stacked GO sheets. Consequently, the internal stress that is induced by interlayer expansion could largely shift the resonant frequency down, leading to the nonlinear frequency response. In the similar way, the lag of desorption between the central region and edges of the underlying GO sheets would result in a high hysteresis [22].

## 4. Conclusions

In summary, a highly sensitive linear pMUT array based humidity sensor functionalized with a GO thin film was designed, fabricated, and characterized. The impedance and phase analysis was used to analyse the frequency characteristics of the pMUT before and after the deposition of the GO film and investigate the humidity-sensing properties of the pMUT humidity sensor. The developed sensor exhibited excellent humidity sensitivity (719 Hz/% RH) and relative sensitivity (271 ppm/% RH) in a wide sensing range of 10% to 90% RH. Meanwhile, rapid response and recovery, small hysteresis, good stability, and a linear frequency-temperature response were also achieved. A possible humidity-sensing mechanism of the GO film coated pMUT was proposed according to the tested experimental results. This work demonstrates that the GO thin film functionalized pMUT is a good candidate for humidity detection and holds potential in the simultaneous detection of multiple parameters, owing to its highly integrated array structure.

## Figures and Tables

**Table 1:** Comparison of different resonant humidity sensors.

Device Type	Sensing Material	*f*_0_ (MHz)	Range (% RH)	Response/Recovery	Hysteresis	*S* (kHz/% RH)	*S_R_* (ppm/% RH)
SAW [19]	GO	392	10–90	22/8 s	3%	11.61	29.62
SAW [29]	CeO_2_/PVP	1560	11–95	16/16 s	-	27.381	17.55
FBAR [9]	ZnO	1431.165	22–82	-	-	8.5	5.94
FBAR [30]	GO	1247	0–83	~4/2 min	-	6.6265	5.31
QCM [22]	GO/PEI	10	11.3–97.3	53/18 s	1%	0.0273	2.73
QCM [26]	GO	10	6.4–97.3	45/24 s	~8%	0.0287	2.87
Cantilever [11]	GO	2.12	10–90	30/10 s	~7%	0.13125	61.91
cMUT [12]	Mesoporous silica	47.4	0–80	~70/14 s	<1%	2.19	46.2
pMUT	GO	2.65285	10–90	<78/54 s	<4%	0.71937	271.17
